# Practices and attitudes towards tuberculosis and latent tuberculosis infection screening in people living with HIV/AIDS among HIV physicians in Japan

**DOI:** 10.1186/s12981-022-00487-8

**Published:** 2022-12-03

**Authors:** Lisa Kawatsu, Noriyo Kaneko, Mayumi Imahashi, Keisuke Kamada, Kazuhiro Uchimura

**Affiliations:** 1 Department of Epidemiology and Clinical Research, The Research Institute of Tuberculosis, 3-1-24, Matsuyama, Kiyose City, Tokyo, Japan; 2 Graduate School of Nursing, Nagoya City University, 1 Kawasumi, Mizuho-Machi, Mizuho, Nagoya, Aichi Japan; 3 Laboratory of Infectious Diseases, Department of Infectious Diseases and Immunology, National Hospital Organization Nagoya Medical Center, 4-1-1 Sannomaru, Naka-Ku, Nagoya City, Aichi Japan

**Keywords:** Tuberculosis, Latent tuberculosis infection, Screening, HIV/AIDS, Japan

## Abstract

**Background:**

Tuberculosis (TB) continues to be the leading cause of death for people living with HIV/AIDS (PLHIV), and HIV is the strongest known risk factor for progression to active TB disease for persons with latent TB infection (LTBI). Screening for active TB and LTBI, and TB preventive therapy (TPT) is recommended, however, clinical practices regarding LTBI screening for HIV positive population have not been uniform, resulting in low rates of LTBI screening and TPT uptake, in both low and high TB-burden countries. We sought to explore the practices and attitudes towards TB and LTBI screening in PLHIV among HIV physicians in Japan.

**Methods:**

We conducted a cross-sectional survey whereby an on-line questionnaire was administered to physicians who are currently, or have the experience of, providing care and treatment for PLHIV in Japan.

**Results:**

The questionnaire was sent to a total of 83 physicians, of which 59 responded (response rate; 71.1%). 52.5% (31/59) conducted routine screening and 44.0% (26/59) conducted selectively screening for active TB among HIV/AIDS patients. As for LTBI, 54.2% (32/59) conducted routine screening and 35.6% (21/59) conducted selective screening for LTBI among PLHIV. “T-SPOT only” was the most frequently used method of screening (n = 33), followed by “QFT only” (n = 11). Criteria for LTBI screening included TB burden in the country of birth of the patient, previous contact with a TB patient, and CD4+ cell count. 83.1% (49/59) either “always” or “selectively” offered TPT to PLHIV diagnosed with LTBI, and among the 49 respondents who did provide TPT, 77.6% (38/49) chose 9-months isoniazid as their first choice. None chose regimen including rifampicin.

**Conclusions:**

Our study revealed that practices regarding TB and LTBI screening and treatment for PLHIV among HIV physicians were mixed and not necessarily in accordance with the various published guidelines. Building and disseminating scientific evidence that takes into consideration the local epidemiology of TB and HIV in Japan is urgently needed to assist physicians make decisions.

**Supplementary Information:**

The online version contains supplementary material available at 10.1186/s12981-022-00487-8.

## Background

Tuberculosis (TB) continues to be the leading cause of death for people living with HIV/AIDS (PLHIV), and HIV is the strongest known risk factor for progression to active TB disease for persons with latent TB infection (LTBI), despite the massive scale-up of combined antiretroviral therapy (cART) in the recent years [1]. According to the most recent report from the WHO, in 2021, 6.4 million incidence cases of TB were reported, of which 6.7% were reported among PLHIV [2]. Furthermore, a systematic review of postmortem studies of global AIDS-related deaths in adults reported TB to be the primary cause of death in 37.2%, and TB being undiagnosed prior to death in 45.8% of cases [3].

Japan is a low-TB burden country, with 11,519 cases newly notified in 2021, giving a notification rate of 9.2 per 100,000 population [4]. TB treatment is provided by TB hospitals, which are specifically designated by the Ministry of Health, Labour and Welfare. With regards to HIV/AIDS, Japan has historically had a low HIV prevalence; in 2021, 742 HIV and 315 AIDS cases were newly reported, largely among men who have sex with men [5, 6]. Free and anonymous HIV testing is offered at public health centers, as well as non-governmental organizations and patient support groups across Japan. HIV care and treatment is provided at specialist hospitals, again designated by the Ministry of Health, Labour and Welfare, of which there are 382 in Japan, and there are a total of 365 physicians who are certified to provide HIV treatment [7, 8]. There are various social welfare systems which may be used to partially cover for the treatment cost, however, it is not totally free. Considering the low HIV prevalence, TB-HIV co-morbidity has therefore been thought to be of less importance compared to other similarly industrialized countries, with *Pneumocystis jirovecii* and candidiasis contributing to approximately 80%, while TB only to 6%, of all reported opportunistic infections in Japan [5]. However, the proportion of those co-infected with TB is much higher among foreign-born than Japan-born PLHIV (15.2% vs 4.5%)—furthermore, while the number of new cases of both TB and HIV/AIDS has continued to decline, the burden of both diseases has increased among foreign-born persons [9].

In order to ensure early detection and timely treatment of TB among PLHIV, major international guidelines have recommended routine screening for active TB, LTBI and TB preventive therapy (TPT) for persons diagnosed with LTBI. In Japan too, several guidelines exist simultaneously—however, the details are very limited. The main three guidelines on HIV/AIDS treatment and care which mention TB and LTBI management in Japan are compared with selected international guidelines in Box 1.

**Table:** Box: Comparison of main Japanese and selected international guidelines on TB/LTBI screening for PLHIV.

Main guidelines in Japan	Selected international guidelines
*HIV kansenshou - chiryou no tebiki. Dai 25 han* [10] (“Treatment guideline on HIV infection. 25th edition”. Research Group for Therapy of HIV Infection. 2021, http://www.hivjp.org/guidebook/)	*EACS Guidelines Version 11.1* [13] (European AIDS Clinical Society. 2022, https://eacs.sanfordguide.com/)
*Ko-HIV chiryou-guideline* [11] (“Guideline on combined anti-retro virus therapy”. 2021, https://hiv-guidelines.jp/index.htm)	*Guidelines for the prevention and treatment of opportunistic infections in adults and adolescents with HIV* [14] (National Institutes of Health, Centers for Disease Control and Prevention, and the HIV Medicine Association of the Infectious Disease Society of America. 2022, https://clinicalinfo.hiv.gov/en/guidelines/hiv-clinical-guidelines-adult-and-adolescent-opportunistic-infections/whats-new)
*HIV kansensho shindan, chiryoum kango manual* [12] (“Manual on diagnosis, treatment and care for HIV positive patients. 2020, https://www.hok-hiv.com/for-medic/download/manual.html)	*BHIVA guidelines for the management of tuberculosis in adults living with HIV 2018 (2022 interim update)* [15] (British HIV Association. 2020, https://www.bhiva.org/TB-guidelines)
On screening for active TB: • No specific mention	On screening for active TB: • Recommends routine chest X-ray (CXR) in persons from high TB prevalence populations [13]
On screening for LTBI: • Recommends IGRA for patients whose CD4+ cell count has recovered to above 200 cells/μL after initiating cART [10]	On screening for LTBI: • Mentions that various national guidelines consider risk factors such as ethnicity, CD4+ cell count and cART usage to define indication for LTBI screening [13] • Recommends LTBI screening for all PLHIV at the time of HIV diagnosis, regardless of their epidemiological risk of TB exposure [14] • Recommends LTBI screening for PLHIV from countries with high and medium TB incidence, regardless of their CD4+ cell count and receipt of cART, and PLHIV from low TB incidence countries, if with additional TB risk factors [15]
On LTBI screening methods: • Mentions IGRA as a test for TB infection, stating that IGRA is more sensitive than TST, and T-SPOT TB is more sensitive than QuantiFERON [11, 12]	On LTBI screening methods: • Recommends either TST or IGRA depending on availability and local standard of care [13] • Both TST and the approved IGRAs are considered appropriate for TB screening among PLHIV in the US. The routine use of both TST and IGRAs in a single patient is not recommended [14] • Recommends IGRA over TST [15]
On LTBI treatment: • Recommends TPT (9 months of isoniazid or 4 months of rifampicin) if diagnosed with LTBI [12]	On LTBI treatment: • Recommends TPT if TST > 5 mm or positive IGRA or close contacts to persons with sputum smear positive tuberculosis, after active TB is excluded (6 or 9 month of isoniazid + pyridoxine or 4 months of rifampicin or 3 months of isoniazid, rifampicin + pyridoxine) [13] • Recommends TPT if positive TB screening test, after active TB is excluded (3 months rifapentine and isoniazid + pyridoxine, or 3 months of isoniazid and rifampicin + pyridoxine, or 6 or 9 months of isoniazid + pyridoxine, or 4 months of rifampin, or 1 month of isoniazid and rifapentine + pyridoxine) [14] • Recommends TPT if positive IGRA, after active TB is excluded (6 months isoniazid + pyridoxine, or 3 months of rifampicin and isoniazid + pyridoxine) [15]

*TB* tuberculosis, *LTBI* latent tuberculosis infection, *PLHIV* people living with HIV, *IGRA* interferon-gamma release assay, *TST* tuberculin skin test, *cART* combined anti-retroviral therapy, *TPT* tuberculosis preventive therapy

As for screening for active TB, globally, for high-burden settings, WHO has been recommending routine screening with the WHO four-symptom screen (W4SS; comprising of current cough, fever, night sweat, and weight loss) [16]. If the W4SS is positive, it is recommended that the patient receives a WHO recommended molecular rapid diagnostic tests (e.g. Xpert MTB/RIF or Xpert MTB/RIF) [17]. For low-burden settings, however, where it is expected that the majority of PLHIV are receiving cART, WHO does not specify the frequency with which PLHIV should receive TB screening [18].

Thus, guidelines in low-incidence countries have varied in their recommendations—for example, in a multi-country study on TB/HIV collaborative policies in European countries, in which 47 countries participated, it was reported that 21 (62%) recommended screening all PLHIV for TB, while 13 (38%) recommended selective screening. The criteria for selective screening included previous contact with a TB case, TB symptoms, previous history of TB and low CD4+ cell count (< 350 cells/μL) [19]. The most recent guideline from the European AIDS Clinical Society has recommended routine screening using chest X-ray (CXR) for PLHIV from high TB prevalence populations [13]. On the other hand, none of the three Japanese guidelines gave clear recommendations regarding screening for active TB.

With regards to LTBI screening, while all three international guidelines have delivered clear recommendation on when to consider LTBI screening for PLHIV [13–15], only one of the three Japanese guidelines has mentioned LTBI screening [10]. The currently available screening tools for LTBI are tuberculin skin test (TST) and interferon-gamma release assays (IGRA), of which there are two kinds, QuantiFERON^®^-TB (QFT; Cellestis, Australia) and T-SPOT.TB^®^-(T-SPOT, Oxford Immunotec, UK) [20, 21].Studies have reported sensitivity estimates of TST, QFT and T-SPOT to be 71%, 61% and 72%, respectively [22, 23], however, a large European cohort study concluded that superiority of IGRA over TST for PLHIV could not be determined [24]. This has led to international guidelines varying on their recommendations, with some recommending either TST or IGRA, while the guideline from UK recommending IGRA over TST. In Japan, two guidelines have mentioned IGRA as a test for TB infection.

As for LTBI treatment, again, while all three international guidelines have recommended several treatment options, which have included short i.e., 3 months, regimen [13–15], only one of the three Japanese guidelines has mentioned and recommended specific treatment regimen [12].

However, despite these guidelines, to date, clinical practices regarding LTBI screening for PLHIV have not been uniform, resulting in low rates of LTBI screening and TPT uptake, in both low and high TB-burden countries [25, 26]. In Japan too, it is not known to what extent the guidelines are being followed on the ground, and what the actual practices of HIV physicians are on TB and LTBI screening. We thus sought to explore the practices and attitudes towards TB and LTBI screening and treatment in PLHIV among HIV physicians, as a first step towards building evidence which may be used to inform screening and treatment policies for TB and LTBI for PLHIV in Japan.

## Method

We conducted a cross-sectional survey whereby an on-line questionnaire was administered to physicians who are currently, or have the experience of, providing care and treatment for PLHIV at the aforementioned designated hospitals for HIV/AIDS care and treatment in Japan. Designated hospitals for HIV/AIDS were identified from the list [14], and potential participants were selected purposively, and were approached directly by e-mail, or via snow-ball sampling method. The questionnaire was created using a survey software, Questant, powered by MACROMILL Inc., and comprised of 29 closed and open ended questions. An invitation containing the link to the questionnaire, together with the description and the purpose of the study, was sent by email to the potential participants. The participants were asked to access the questionnaire between September 1st and October 31st, 2021.

The first section of the questionnaire asked about the physician’s socio-demographic information and experience of providing HIV care and treatment. The second section asked about screening practices for active TB, and the third about screening practices for LTBI and knowledge regarding guidelines for LTBI screening and TPT for PLHIV in Japan. The final section asked about treatment practices for those diagnosed with LTBI. The questionnaire was designed so that participants could proceed and complete the survey even if they left some questions unanswered. The questionnaire was piloted with two physicians and was revised as necessary, prior to commencement of the survey. A translated version of the questionnaire is available as a Additional file 1: Appendix 1. The data was analyzed using SPSS (version 22.0). Descriptive statistics were used to calculate the overall response rate and frequencies for each question. The study protocol was reviewed and approved by the Institutional Review Board of the Research Institute of Tuberculosis, Japan Anti-Tuberculosis Association (reference number: RIT/IRB 2021-05).

## Results

The questionnaire was sent to a total of 83 physicians, of which 59 responded (response rate; 71.1%). Those question items whereby the participants did not give response were categorized as “No Answer”. The socio-demographic characteristics of the respondents are summarized in Table 1.

**Table 1:** Characteristics of the respondents (n = 59)

	n	%
Total	59	100.0
Sex		
Male	52	88.1
Female	7	11.9
Age groups (years old)		
< 39	24	40.7
40–49	22	37.3
50–59	10	16.9
60–69	3	5.1
Currently working in HIV care		
Yes	21	35.6
No	12	20.3
No answer	26	44.1
Years of working in HIV care		
1–2 years	6	10.2
3–5 years	18	30.5
6–10 years	17	28.8
11 years or more	18	30.5
No. new HIV/AIDS patients seen at the participant’s institution a year		
0–5 cases	11	18.6
6–10 cases	10	16.9
11–15 cases	9	15.3
16–20 cases	4	6.8
21 cases or more	25	42.4

### Screening practices for active TB

**Fig. 1:**
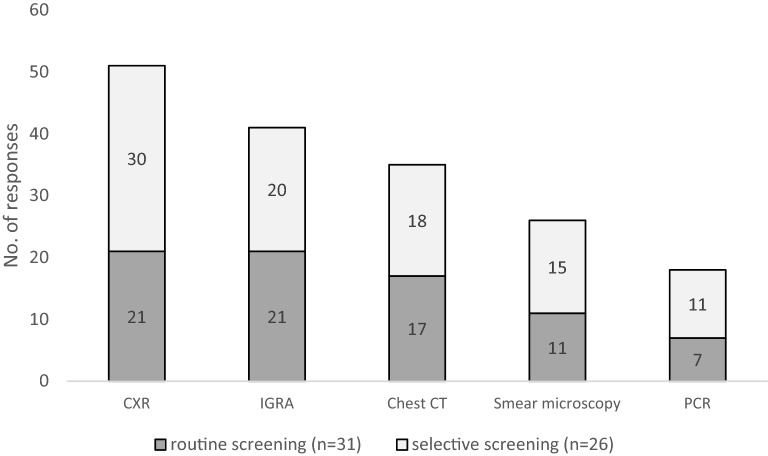
Method of screening for active TB (multiple responses allowed). *TB* tuberculosis, *CXR* chest X-ray, *CT* computed tomography, *PCR* polymerase chain reaction, *TST* tuberculin skin test, *IGRA* interferon-gamma release assays

52.5% (31/59) conducted routine screening and 44.0% (26/59) conducted selectively screening for active TB among PLHIV. CXR was the most frequently used method of screening (n = 51), followed by interferon-gamma release assays (IGRA) (n = 41) and chest computed tomography (CT) (n = 35). As a tool for routine screening, CXR and IGRA were the two most frequently given choice (n = 21), followed by chest CT (n = 17) (Fig. 1).

Among the 26 respondents who conducted selective TB screening, criteria for screening were further asked. The most frequently given criterion was “respiratory symptoms” (n = 26), followed by “country of birth of the patient” (n = 21) and “history of previous TB treatment” and “other socio-economic background” (n = 19) (see Table 2).

**Table 2:** Criteria for screening for active TB (multiple responses allowed, n = 26)

Screening criteria for active TB	n	%
Respiratory symptoms	26	100
Country of birth of the patient	21	80.8
History of previous TB treatment	19	73.1
Other socio-economic background	19	73.1
History of contact with a TB case	18	69.2
Other non-respiratory symptoms	15	57.7
Others	2	7.7

*TB* tuberculosis

However, the most frequently given combination of screening criteria was “all” (n = 10), followed by “respiratory symptoms, history of previous TB treatment, history of contact with a TB case, and country of birth” (n = 7). Furthermore, among the 57 that either conducted routine or selective screening for active TB, two responded that the screening was an institutional policy, whereas 24 responded that the decision was entrusted to individual physicians. 31 did not provide response. As for the 2 respondents who did not conduct screening for active TB, one respondent gave “not having a chest X-ray machine”, and the other “at that time, IGRA was not available and screening using TST was not considered appropriate” as their reasons.

### Screening practices for LTBI

**Fig. 2:**
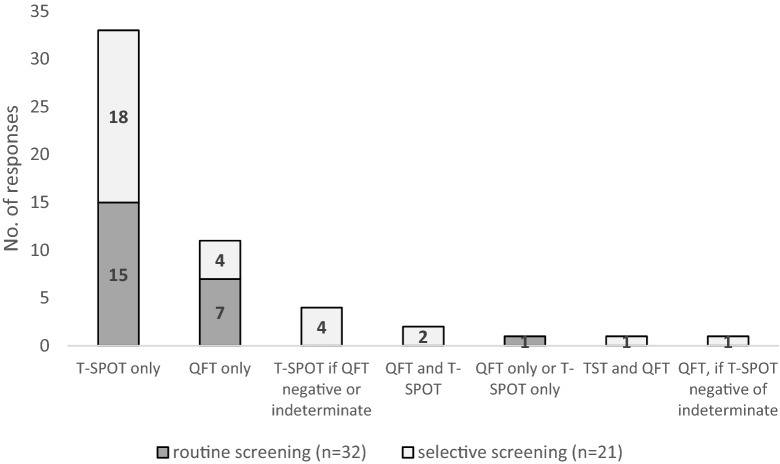
Method of screening for LTBI (multiple responses allowed). *TST* tuberculin skin test, *QFT* QuantiFERON

54.2% (32/59) conducted routine screening and 35.6% (21/59) conducted selective screening for LTBI among PLHIV. “T-SPOT only” was the most frequently used method of screening (n = 33), followed by “QFT only” (n = 11), and “T-SPOT, if QFT negative or indeterminate” (n = 4). As a tool for routine screening, “T-SPOT only” was the most frequently given choice (n = 15), followed by “QFT only” (n = 7) (Fig. 2).

Criteria for screening among the 21 respondents who conducted selective LTBI screening for PLHIV are summarized in Table 3. The most frequently given criterion was the “TB burden in the country of birth of the patient” (n = 19). However, when further asked what exactly defined the “TB burden” which would lead to the decision to conduct LTBI screening, 17 responded “patient coming from a TB high- or middle-burden country as defined by WHO”, 10 responded “patient coming from Southeast Asian country”, and another 9 responded “patient coming from African country”. The second most frequently given response was a history of contact with a TB case (n = 17), followed by a history of previous TB treatment (n = 16).

**Table 3:** Criteria for screening for LTBI (multiple responses allowed, n = 21)

Screening criteria for LTBI	n	%
TB burden in the country of birth of the patient	19	90.5
TB incidence ≥ 40/100,000	0	0.0
TB incidence ≥ 20/100,000	4	19.0
TB “high-burden” according to WHO definition	2	9.5
TB “high- and middle-burden” according to WHO definition	17	81.0
COB is from African region	9	42.9
COB is from Southeast Asian region	10	47.6
COB is from South American region	5	23.8
Others	1	4.8
History of contact with a TB case	17	81.0
History of previous TB treatment	16	76.2
CD4+ cell count (cells/μL)	7	33.0
≦50	0	0.0
51–100	0	0.0
101–200	4	19.0
201–350	1	4.8
351–500	0	0.0
Others	2	9.5
Duration of cART treatment	1	4.8
6 months or less	1	4.8

*LTBI* latent tuberculosis infection, *TB* tuberculosis, *COB* country of birth, *cART* combined anti-retroviral treatment

Of the 6 respondents who answered they did not conduct LTBI screening, the most frequently given reason was that they felt that the “risk of TB was low among HIV patients who visited their hospital” (n = 4), followed by “LTBI testing not being available at their hospital” (n = 3), and they felt that they “could not trust the results of LTBI test” (n = 2).

### Knowledge and attitudes towards guideline on LTBI testing

The respondents were also asked about their knowledge and attitudes towards Japanese guidelines on HIV care and treatment in Japan. Firstly, the respondents were asked about the guideline published by the Japanese Society for AIDS Research, which recommended “…testing for TB infection, using methods such as IGRA, when CD4+ cell counts have improved to ≥ 200 cells/μL after initiating cART”. 84.7% (50/59) agreed with the recommendation, of whom 42.4% (25/59) were already aware of the guideline at the time of the study. On the other hand, 8.5% (5/59) answered that they were aware of but disagreed with the guideline, and 6.8% (4/59) answered that they were unaware of and disagreed with the recommendation.

Secondly, regarding the method of LTBI testing, the respondents were asked whether they agreed with the two guidelines, one published by Hokkaido University Hospital, one of the designated hospitals for HIV and AIDS treatment in Japan, and another by a government-funded research group, both of which stated that “T-SPOT TB has higher sensitivity than QnatiFERON (QFT-3G)”. The respondents were asked whether they were aware of the guideline, and also agreed that T-SPOT should be preferred over QFT-3G. 61.0% (36/59) agreed that T-SPOT should be preferred over QFT-3G, of whom 11.9% (7/59) answered that they were already aware of the guideline at the time of the study. On the other hand, 8.5% (5/59) answered that they were aware of but disagreed with the guideline, and 30.5% (18/59) answered that they were unaware of and disagreed with the guideline.

### Practices on LTBI treatment

Table 4 summarizes the treatment practice for LTBI. The respondents were asked if PLHIV were diagnosed with LTBI, whether patients would be offered TPT, and if so, the first priority regimen. 83.1% (49/59) either “always” or “selectively” offered TPT to patient diagnosed with LTBI, and among the 49 respondents who did provide TPT, 77.6% (38/49) chose 9-months isoniazid as their first choice. None chose regimen including rifampicin.

**Table 4:** LTBI treatment practice for PLHIV and preferred regimen

	n	Of which 6H	Of which 9H
Yes, TPT is routinely offered to PLHIV with LTBI	26	3	23
Yes, TPT is selectively offered to PLHIV with LTBI	23	8	15
No, TPT is not offered to PLHIV with LTBI	1	NA	NA
I do not know	9	NA	NA
Total	59	11	38

*H* isoniazid, *TPT* tuberculosis preventive therapy, *PLHIV* people living with HIV, *LTBI* latent tuberculosis infection

Among those who responded that they “selectively” offered TPT, they were further asked to give, in free texts, possible factors that they would consider when making the final decision. The responses included “risk of TB/TB infection prior to diagnosis”, “possible drug interactions”, “history of interrupting previous TB treatment”, “CD4+ cell count”, “overall physical and mental status of the patient”, “age of the patient”, “expected adherence” and “spot count of T-SPOT”. The one respondent who answered that he/she did not provide TPT, gave “concern for drug interaction” and “concern for drug resistance” as the reason.

### Knowledge and attitudes towards guidelines on LTBI treatment

Respondents were asked whether they were aware and agreed with the recommendation given by the guideline published by the Hokkaido University Hospital, stating that “HIV patients who have been diagnosed with LTBI by IGRA should proactively be offered treatment by 9-months isoniazid, and in case isoniazid is contraindicated, 4-months rifampicin”. 84.7% (50/59) agreed with the recommendation, of whom 35.6% (21/59) were already aware of the guideline at the time of the study. On the other hand, 8.5% (5/59) answered that they were aware of but disagreed with the guideline, and 6.8% (4/59) answered that they were unaware of and disagreed with the guideline.

Finally, respondents were asked if they would have any plan to actively recommend TPT for PLHIV diagnosed with LTBI. 86.4% (51/59) answered “yes”, of whom 40 would recommend a 9-months isoniazid regimen, 5 would recommend a 6-months isoniazid regimen, 2 a 4-months rifampicin regimen, and 4 a shorter regimen, when and once it is officially approved in Japan.

## Discussion

This study is the first to have explored the practices and attitudes of HIV physicians regarding TB and LTBI screening and practice in Japan in the recent years. The results have indicated that physicians varied, not only in their practices and attitudes but also opinions regarding various guidelines.

### Screening for active TB and LTBI

While none of the Japanese guidelines gave any specific recommendations regarding TB screening for PLHIV, in our study, 52.5% in fact conducted routine screening and a further 44.0% conducted selective screening for active TB, and their decisions were based on several criteria. On the other hand, the proportions of those conducting screening for LTBI, either routinely or selectively, were smaller compared to those conducting screening for active TB. The criteria to consider screening were in line with those recommended by international guidelines, including TB burden and other epidemiological risk factors for TB. These results may suggest that testing for active TB for PLHIV is more or less considered a routine practice, while awareness and knowledge towards LTBI testing and treatment may be limited among HIV physicians in Japan. This is partially due to Japan’s unique historical and social tradition of medicine, which for long has been biased towards promoting and training non-generalist, i.e., specialist care [27]. This has meant that HIV and AIDS patients are generally taken care by infectious disease or hematology specialists, while TB is taken care by respiratory physicians. Lack of coordination and communication between different specialist, even within the same institution, has been raised as a long standing issue by several studies from Japan [28, 29].

With regards to screening methods, the majority of the respondents chose IGRA based single-testing method (either T-SPOT only or QFT only). Considering that BCG vaccination is still routinely administered, and TST is typically only now used as a test of TB infection for very small children in Japan, it is understandable that none chose TST only. However, evidence regarding effectiveness of T SPOT and QFT are not conclusive—systematic review and meta-analysis studies have reported higher pooled sensitivity for T-SPOT than QFT, however, the difference was not statistically significant [30, 31]. A more recent study from the US has suggested that T-SPOT showed higher positive predictive value than QFT and TST, which “may make it preferable for screening PLHIV with relatively high CD4+ cell counts in low risk settings”. However, the study also concluded that additional prospective studies are needed to evaluate the incremental cost-effectiveness of different strategies [32]. We were unable to determine the reasons for the preference towards QFT among the study participants—however, some possible reasons include QFT being introduced to Japan earlier than T-SPOT (QFT in 2005, T-SPOT in 2012) and thus the former perhaps more well-known, and several Japanese studies indicating higher performance of QFT compared with T-SPOT [33, 34].

### LTBI treatment

As for LTBI treatment, international guidelines have clearly maintained that PLHIV who have a positive test for LTBI should be offered TPT, after active TB is ruled out [13–15]. The guideline from the US further recommended LTBI treatment for PLHIV who reports close contact with infectious TB case, regardless of TB screening test results [14]. Various treatment options are currently recommended. Recently short-course rifamycin-based treatment are preferred over isoniazid due to better adherence and treatment and completion rates, as demonstrated in several studies [35–38]. However, in Japan, only one guideline specifically recommended TPT, with 9-months of isoniazid or, if isoniazid is not available, 4-months of rifampicin [12]. TPT was not even mentioned in the other two guidelines. The majority of the respondents in our study preferred 9-months isoniazid, despite showing concern for poor adherence. None prescribed 4-months rifampicin, and only 2 expressed their future intention. Rifampicin is a potent inducer of the cyto-chrome P450 hepatic enzyme system and is thus contraindicated with HIV protease inhibitors. Furthermore, there is a theoretical risk of drug resistance if rifampicin monotherapy is administered before ruling out active TB. The general reluctance towards using rifampicin may also be due to the physicians’ concern for the possibility of having to change the course of cART and thereby confuse patients and/or aggravate adherence.

### Adherence to guidelines

Despite numerous guidelines, it has also been reported in several studies, that practices on the ground often differ substantially from what is recommended [39–41], and often a significant proportion of PLHIV do not receive screening for LTBI [37, 40, 42]. Uptake of LTBI screening in industrialized countries have ranged from 20% in Belgium, to 68.8% in US [43]. In our study, 54.2% of the participants conducted routine, and 89.9% selective screening for LTBI.

Several studies have explored the possible reasons for non-adherence to recommendations on LTBI screening and treatment and pointed out to perceived low accuracy of LTBI tests or lack of sufficient evidence [37, 40], perceived low risk for LTBI among PLHIV in the local settings or low risk among those on cART [39], fear of potential side effect and drug interactions and poor compliance [39, 40]. One study even reported explicitly negative attitude towards guidelines, due to lack of end-user involvement in the development of such guidelines [39].

Our study did not explore the potential barriers to physicians adhering to guidelines in detail, and the number of respondents who did not conduct LTBI testing and treatment was quite small. It is therefore probably not appropriate to generalize the potential barriers to LTBI testing and treatment which were raised by our respondents. On the other hand, as mentioned earlier, in Japan, TB and HIV/AIDS treatment and care are provided at specialist hospitals, each designated by the Ministry of Health, Labour and Welfare and they do not necessarily overlap. In fact, there is a very limited number of facilities that are designated as both TB and HIV/AIDS hospitals—thus, there might simply be a lack of awareness on TB and LTBI, and recent developments thereof among HIV physicians in Japan. Opportunities to exchange expert opinions between TB and HIV/AIDS specialists are encouraged.

What also became apparent from out study was that the respondents tended to rely on several risk factors to guide their decisions, which were not mentioned in the Japanese guidelines or necessarily supported by strong evidence. What is therefore needed are epidemiological studies to identify and evaluate possible risk factors for LTBI among PLHIV in Japan, so that evidence-based risk assessment scale may be developed to assist physician in their clinical decisions regarding whether or not to apply LTBI screening and treatment. Discussions and evidence gathering are also urgently needed regarding shorter treatment regimens, taking into consideration the epidemiological situation of TB and HIV in Japan.

## Limitations

Our study is not without limitations. Firstly, the participants were not selected randomly and the number of participants was limited to 59. There is therefore the possibility that the responses may not be generalizable. On the other hand, a previous study has suggested that experience of providing HIV treatment and care may be disproportionately skewed towards quite a limited number of hospitals, which are well-known and popular among PLHIV [28, 44]. The participants in this study included physicians working in two main urban cities, Tokyo and Osaka, where the majority of PLHIV in Japan concentrate, as well as physicians working at core regional hospitals. The responses may be thought to be representative, at least of those physicians who are routinely involved in providing HIV care and treatment.

Secondly, the study comprised of limited number of, and simple questions, to encourage participation of physicians with busy schedule. We were therefore not able to explore in detail the reasons for the choice of the participants. The study was however intended to be exploratory in nature, and it is hoped that more detailed studies will follow.

## Conclusion

Our study revealed that practices regarding TB and LTBI screening and treatment for PLHIV among HIV physicians were mixed and not necessarily in accordance with the various published guidelines. Building and disseminating scientific evidence that takes into consideration the local epidemiology of TB and HIV in Japan is urgently needed to assist physicians make decisions.

## Supplementary Information

**Additional file 1: Appendix 1.** On-line questionnaire survey.

## Data Availability

Raw questionnaire data is available upon request.
