# Supplementary material for: Practices and attitudes towards tuberculosis and latent tuberculosis infection screening in people living with HIV/AIDS among HIV physicians in Japan
Source: AIDS Res Ther. 2022 Dec 3;19:60. doi: 10.1186/s12981-022-00487-8 (PMC9719667; doi:10.1186/s12981-022-00487-8)
Supplement: Supplementary file 1 — Additional file 1: Appendix 1. On-line questionnaire survey. [file 12981_2022_487_MOESM1_ESM.docx]

Appendix 1. On-line questionnaire survey

Note: The questionnaire was conducted in Japanese. The questions were translated into English for the purpose of publication.

| **Question** | **Response** |
| --- | --- |
| 1. What is your sex? | □Male □Female |
| 1. What is your age? | □29 years old or younger □30-39 □40-49 □50-59 □60-69 |
| 1. Are you currently involved in providing HIV care and treatment? | □Yes □No |
| 1. If so, what is the type of health facility which you currently work for? | □Regional AIDS hospital　□Core AIDS hospital  □AIDS rehabilitation hospital　□Others |
| If others, please specify. | Free text |
| 1. For how many years have you been involved in providing HIV care and treatment? | □1-2 □3-5 □6-10 □11 or more |
| 1. In which department do you work (or have you worked, when you were providing HIV care and treatment)? | □Infectious diseases　□Hematology  □Respiratory medicine　□Others |
| 1. How many new HIV/AIDS patients do you see on average in a year? | □1-5 □6-10 □11-15 □16-20　□21 or more |
| 1. Do you conduct screening for active TB for new HIV/AIDS patients? | □Yes, always　□Yes, under certain conditions  □No |
| 1. If yes, what do you use? (please select all that applies) | □CXR □Chest CT □smear microscopy □PCR　 □TST □IGRA □Others, specify |
| 1. If yes, what is/are the criterion/ia upon you base your decision whether to conduct TB screening or not? (please select all that applies) | □respiratory symptoms □other symptoms  □previous history of TB  □contact with an active TB patient  □TB burden in the country of birth of the patient  □Other socioeconomic risks □Others, specify |
| 1. Is the TB screening for new HIV/AIDS patients an institutional policy? | □Yes □No |
| 1. If you do not conduct TB screening for new HIV/AIDS patients, what is/are the reason(s)? | Free text |
| 1. Do you conduct screening for LTBI for new HIV/AIDS patients? | □Yes, always　□Yes, under certain conditions  □No |
| 1. If yes, what do you use? | □TST only □QFT only □T-SPOT only  □QFT of TST negative　 □TST and QFT  □T-SPOT if TST negative □TST and T-SPOT  □T-SPOT if QFT negative or indeterminate  □QFT if T-SPOT negative or indeterminate  □T-SPOT and QFT □Others, specify |
| 1. If yes, what is/are the criterion/ia upon you base your decision whether to conduct LTBI screening or not? (please select all that applies) | □previous history of TB  □contact with an active TB patient  □TB burden in the country of birth of the patient  □CD4+ cell count □time on cART □Others, specify |
| 1. If you use “TB burden in the country of birth of the patient”, what is the threshold to conduct LTBI screening? | □TB incidence ≧ 40/100,000  □TB incidence ≧ 20/100,000  □TB high-burden according to WHO  □TB high and middle-burden according to WHO  □The patient is from a country in Africa  □The patient is from a country in Southeast Asia  □The patient is from a country in South America  □Others, specify |
| 1. If you use “CD4+ cell count”, what is the threshold to conduct LTBI screening? | □Above 101 and below 200  □Others, specify |
| 1. If you use “time on cART treatment”, what is the threshold to conduct LTBI screening? | □Within 6 months  □Others, specify |
| 1. If you do not conduct LTBI screening for new HIV/AIDS patients, what is/are the reason(s)? (please select all that applies) | □I do not think there is a need for TPT.  □I do not trust the results of LTBI screening.  □It is difficult to conduct LTBI screening.  □It is too costly to conduct LTBI screening.  □TPT is not offered at my hospital/clinic.  □I do not think that risk of developing TB is high among our patients.  □Others, specify |
| 1. What is your opinion regarding the recommendation from the *“HIV kansenshou chiryou no tebiki”,* stating that IGRA should be conducted to test for TB infection? | □I knew of the guideline, and I agree.  □I knew of the guideline, but I do not agree.  □I did not know of the guideline, but I agree.  □I did not know of the guideline, and I do not agree. |
| 1. What is your opinion regarding the statement from the *“HIV kansenshou shindan/chiryou/kango manual”,* stating higher sensitivity of T-SPOT over QFT-3G? | □I knew of the guideline, and I agree.  □I knew of the guideline, but I do not agree.  □I did not know of the guideline, but I agree.  □I did not know of the guideline, and I do not agree. |
| 1. Do you offer TPT for PLHIV diagnosed as LTBI? | □Yes, always　□Yes, under certain conditions  □No |
| 1. If yes, what would be the first treatment regimen of choice? | □6H □9H □Others, specify |
| 1. If yes, depending on certain conditions, what factors would you take into consideration? | Free text |
| 1. If you do not offer TPT, what is/are the reason(s)? (please select all that applies) | □Concerns for side-effects  □Concerns for drug interactions.  □Concerns for development of drug-resistance.  □Concerns for drug adherence. |
| 1. What is your opinion regarding the recommendation from the *“HIV kansenshou shindan/chiryou/kango manual,* stating that TPT should be proactively offered to those diagnosed with LTBI”? | □I knew of the guideline, and I agree.  □I knew of the guideline, but I do not agree.  □I did not know of the guideline, but I agree.  □I did not know of the guideline, and I do not agree. |
| 1. Do you have any plans to actively offer TPT to PLHIV diagnosed with LTBI in future? | □Yes  □I think so.  □I do not think so.  □No  □I am not sure. |
| 1. If yes, what would be the first treatment regimen of choice? | □6H □9H □4R □Other shorter regimens, once approved in Japan. |
| 1. If you have any other opinions/comments regarding TB and LTBI screening for PLHIV in Japan, please tell us. | Free text |

CXR: chest X-ray, CT: computed tomography, TST: tuberculin skin test, IGRA: interferon-gamma release assays, PCR: polymerase chain reaction, TB: tuberculosis, TPT: tuberculosis preventive therapy, LTBI: latent tuberculosis infection, H: isoniazid, R: rifampicin
